# The Psychometric Properties of the Smartphone Application-Based Addiction Scale (SABAS)

**DOI:** 10.1007/s11469-017-9787-2

**Published:** 2017-07-18

**Authors:** Sándor Csibi, Mark D. Griffiths, Brian Cook, Zsolt Demetrovics, Attila Szabo

**Affiliations:** 1 Department of Ethics and Social Sciences, University of Medicine and Pharmacy, Târgu Mureș, Romania; 2 Psychology Department, Nottingham Trent University, 50 Shakespeare Street, Nottingham, NG1 4FQ UK; 3 Kinesiology Department, California State University, Monterey Bay, Seaside, CA USA; 4 Institute of Psychology, ELTE Eötvös Loránd University, Budapest, Hungary; 5 Institute of Health Promotion and Sport Sciences, ELTE Eötvös Loránd University, Budapest, Hungary

**Keywords:** Moble phone dependence, Smartphone addiction, Mobile phone addiction, Nomophobia, Social media addiction

## Abstract

The goal of the study was to validate the English version of the Smartphone Application-Based Addiction Scale (SABAS; Csibi et al. 2016), which is a short and easy-to-use tool for screening the risk of smartphone application-based addiction. Another aim was to identify the most frequently used smartphone applications and their perceived importance by the participants. Data were collected online from 240 English-speaking volunteers, aged 18 to 69 years. The instruments used were the SABAS, the Nomophobia Questionnaire (NMP-Q), the Brief Sensation Seeking Scale (BSSS), the Deprivation Sensation Scale (DSS), and the Patient Health Questionnaire (PHQ-9). Participants also ranked the importance of their most frequently used smartphone applications. The six items of the SABAS yielded one component, which accounted for 52.38% of the total variance. The internal reliability of the scale was good (Cronbach’s alpha 0.81). NMP-Q was a significant predictor of SABAS, explaining 17.6% of the total variance. The regression analysis, with SABAS score as the dependent variable and NMP-Q, DSS, PHQ-9, and BSSS scores as predictors, indicated that approximately 47% of the variance in SABAS was accounted for by the predictors (*R* ^2^ = 0.47). The English version of the SABAS appears to be a valid and reliable ultra-brief tool for a quick and easy assessment of smartphone application-based addiction symptoms.

Smartphones allow a permanent online presence for users, and studies have asked the important question concerning which smartphone applications are the most prevalent and engrossing, and the underlying psychological or social mechanisms (Demirci et al. 2015; Jeong et al. 2016; Salehan and Negahban 2013) such as anxiety, depression, or daily dysfunction.

Recent reports on smartphone use have suggested that only a small minority of users display problems including addictive-like symptoms (e.g., Billieux et al. 2015; Elhai et al. 2017a, b; Lopez-Fernandez et al. 2017). A systematic review of 117 papers (Elhai et al. 2017a) concluded that depression severity, anxiety, and stress are associated with problematic smartphone use. Although there are innumerable benefits of smartphones, scholars have discussed several detrimental effects including mental health issues (Elhai et al. 2017a, b), poor physical fitness (Lepp et al. 2015; Rebold et al. 2016), and poor academic performance (David et al. 2015; Lepp et al. 2015) resulting as consequences of excessive use.

The literature describes several commonalities and differences between internet and smartphone misuse, such as having similar symptoms to other forms of addiction (Kuss and Griffiths 2012; Kuss et al. 2014; Lopez-Fernandez 2015). Currently, the terminology describing problematic smartphone use is inconsistent, as evidenced by the use of the various terms including “addiction,” “excessive,” “compulsive,” “compensatory,” and “problematic” (e.g., Kardefelt-Winther 2014a, b; Widyanto and Griffiths 2006). These terms have led to a complex and conceptually composite definition of the behavior that causes functional impairment, lack of control, and/or dysfunctional coping (Long et al. 2016).

Theoretical approaches consider negative/positive reinforcement as a modality of coping, and incentive sensitization as a process of mood enhancement, in time modifying the experiences of “liking” into “wanting” (or needing) a behavior, such as (in the case of smartphone use) checking messages or engaging in social media use (Robinson and Berridge 2008). Scholars have also discussed the role of personality-related variables, such as extraversion, loneliness, anxiety, and impulsivity in the process of ensuring positive emotions and reducing negative ones through smartphone use (Billieux et al. 2015). According to Billieux et al. (2015), the habit of checking notifications may provide such positive emotions through the social reassurance stemming from the behavior of the friends. Authors have described constructs such as the “reassurance seeking” and the “fear of missing out” pathways leading to an excessive use of smartphone including symptoms such as low self-esteem, loneliness, depression, and anxiety (Billieux et al. 2015; Elhai et al. 2016). Other personality-related variables, such as impulsivity and extraversion, drive the individual to sustain constant relationship with others, involving sensation seeking and reward sensitivity in media use and online behavior (Billieux et al. 2015; Hoffner and Lee 2015).

Among media content types, excessive users of social networking, games, and entertainment are more likely to develop addiction symptoms than those who use smartphones for study/work-related purposes. Furthermore, addiction to social media has a significant association with the increase of depressive symptoms and the decrease of psychological well-being (Jeong et al. 2016). Other studies have shown that lower levels of depression are associated with more engagement in social smartphone use (Elhai et al. 2017a, b). However, it is not known whether increased behavioral activation offsets the impact of problematic smartphone use in relation to depression outcomes. According to Billieux et al. (2015), the frequent use of a smartphone cannot necessarily be treated as an addictive behavior.

Researchers suggest that sensation seeking has a genetic basis responsible for dopamine release (Zuckerman and Kuhlman 2000). This personality trait might manifest itself in preference for violent content and multitasking with different types of online media, evidencing the high sensation seekers’ online activity characteristics (Cservenka et al. 2013; Jeong et al. 2016; Kim et al. 2016a, b; Lin et al. 2015; Roser et al. 2016; Velezmoro et al. 2010).

Research has identified a heterogeneous spectrum of problematic use of smart devices for online activities and applications, such as *Facebook* or other forms of socializing in the virtual world (Billieux et al. 2015; Király et al. 2015; Kuss and Griffiths 2011). Specialists in behavioral addictions highlight the importance of generating valid instruments suitable for the screening and the measurement of technology-related addictive behavior (Kardefelt-Winther 2016; Lopez-Fernandez 2017). The literature features several adaptations and generations of new instruments, such as the adapted Smartphone Addiction Scale-Short Version (SAS-SV) in Spanish and Francophone Belgian countries by Lopez-Fernandez (2017). The factor analyses of both the Spanish and Francophone Belgian versions supported the scale’s unidimensionality that explained 49% and 54% of the variance respectively, good construct validity, and excellent reliability. Other smartphone addiction measures have also demonstrated good reliability, including the Smartphone Addiction Inventory (SPAI) in China (Lin et al. 2014) and the Smartphone Addiction Proneness Scale (SAPS) in South Korea (Kim et al. 2014). Studies testing the factorial structure of smartphone assessment instruments (e.g., Lin et al. 2014; Lopez-Fernandez 2017; Pavia et al. 2016; Kwon et al. 2013) have identified several common variables acting as risk factors in smartphone addiction, including time distortion in using a smartphone, compulsivity with a negative impact on daily life activities and relationships, symptoms of tolerance, craving, and withdrawal symptoms.

In the current study, the primary aim was to validate an English version of the original Hungarian version of the Smartphone Application-Based Addiction Scale (SABAS; Csibi et al. 2016). In contrast to the existing instruments, the SABAS is a short and easy-to-use tool for screening the risk for smartphone addiction. A secondary aim was analyze the preferences for smartphone-based applications in relation to SABAS and Nomophobia^1^ Questionnaire (NMP-Q) scores. Finally, the study also aimed to examine the ways in which these choices influence scores reported on deprivation sensation, depression, and sensation seeking variables.

## Methods

### Participants

The participants comprised 240 volunteers, aged between 18 and 69 years (mean age = 25.4 years), 155 males (64.6%) and 85 females (35.4%). They completed an online questionnaire following recruitment via various internet forums (such as university students’ blogs, *CNET*, *LinkedIn*, *Facebook*), especially those related to mobile application discussion groups. By the nature of the study, self-selection was an a priori delimitation (as well as a later limitation). The research was approved by the Research Ethics Board of a large urban university. All the participants read and agreed to informed consent form prior to taking part in the study.

### Instruments

In addition to a brief demographic questionnaire (e.g., gender, age), the questionnaire also included the SABAS (6 items; Csibi et al. 2016; See Appendix 1), the NMP-Q (20 items; Yildirim and Correia 2015), the Brief Sensation Seeking Scale (BSSS, 8 items; Hoyle et al. 2002), the Deprivation Sensation Scale (DSS, 9 items; Robbins and Joseph 1985), and the Patient Health Questionnaire Depression Scale (PHQ-9, 9 items; Kroenke et al. 2001). Participants were also asked about the most frequently used smartphone applications and asking them to rank by their importance (*“Please list the three most important and most used smartphone applications by yourself in a ranked order [i.e., first, second, and third most important]ˮ*).

### Procedure

The responses to the particular question regarding the most often used applications were transformed into quantitative variables according to nine types of applications (apps): (i) traditional mobile communication and phone function, (ii) internet-based communication, (iii) social media, (iv) information, (v) entertainment, (vi) games, (vii) directions and timetable, (viii) lifestyle applications, and (ix) health-related applications. As noted above, the categories were ranked (1, 2, or 3) and scored according to the participant’s responses (scoring 3 if it was the most popular, 2 if it was second, and 1 if it was ranked third).

To fulfill the secondary aim of the study, a two-step cluster analysis of the data was performed. This generated two distinct clusters, the first with 139 participants (57.9%) and the second with 101 participants (42.1%), with the ratio of sizes 1.38. The clusters were generated based on the test scores. Therefore, in the first cluster, the scores were higher on DSS, SABAS, and NMP-Q and lower on PHQ-9 and BSSS scales; in the second, the scores were lower on DSS, SABAS, and NMP-Q and higher on PHQ-9 and BSSS scales (Table 1).

**Table 1:** The size and the score differences of the applied tests in the two clusters

Cluster	Size	BASS	NMP-Q	DSS	PHQ-9	BSSS
1	57.9% (139)	25.26	93.24	12.77	12.90	25.55
2	42.1% (101)	17.90	64.96	23.61	18.04	26.90

The first cluster had low values on DSS (smartphone use without sensation of deprivation) and high values on SABAS and NMP-Q (showing addiction vulnerability), low scores on depression scale (lack of negative feelings), and moderated values on BSSS. The second cluster had the opposite value on every scale, except the BSSS (which had approximately the same value).

Statistical analysis was performed with PASW 18 (SPSS, Chicago, IL). To explore the underlying factor structure of the SABAS, an exploratory factor analysis (EFA) was performed on the data. Internal consistence reliability using Cronbach’s alpha coefficient was examined. A correlation coefficient was computed to assess the relationship between the scores of the participants on the SABAS, DSS, NMP-Q, PHQ-9, and BSSS. Two-step cluster analysis was used to distribute the sample according to addiction vulnerability. For the analysis of the differences between the two clusters, ANOVA and Mann-Whitney tests were performed.

## Results

Theoretically, the SABAS was expected to assess a single construct (i.e., the level of smartphone addiction). Therefore, the six items of the scale were subjected to factor analyses using the principal component analysis method. Each of the six items of the SABAS was significantly correlated with all the other items in the scale (*p* < 0.01), supporting factorability. The Kaiser-Meyer-Olkin (KMO) measure of sampling adequacy was 0.84. The Bartlett’s test of sphericity was significant (*χ* ^*2*^ (15) = 426.87, *p* < 0.01). Furthermore, the diagonals of anti-image correlation matrix were all over 0.80 supporting the inclusion of each item in the factor analysis. Finally, the commonalities were mostly above 0.40, further confirming that each item shared some common variance with the other items. Given these indicators, a principal axis factoring was performed with the six items of the SABAS. One factor (i.e., a single component, eigenvalue = 3.14) was identified and accounted for 52.38% of the total variance (see Table 2).

**Table 2:** Total variance of the SABAS items

Total variance explained			
Factor	Initial eigenvalues		
	Total	% of variance	Cumulative %
1	3.13	52.29	52.29
2	0.76	12.80	65.10
3	0.66	11.02	76.12
4	0.60	10.13	86.25
5	0.44	7.33	93.58
6	0.38	6.42	100.00

Extraction method: principal axis factoring

The internal reliability of the scale was examined using Cronbach’s alpha and demonstrated good reliability (Cronbach’s alpha = 0.81; mean score = 22.16; std. deviation = 5.69). The Cronbach’s alpha value did not change significantly when one item was deleted suggesting good internal consistency. The congruent validity of the test also appeared to be good. The correlation with the 20-item NMP-Q (Yildirim and Correia 2015) was statistically significant (*r* = 0.63, *p* < 0.01). A significant (negative) correlation (*r* = −0.60, *p* < 0.01) was also found with the nine-item DSS (Robbins and Joseph 1985). The other two scales applied in the present study also showed significant negative correlations with the SABAS (see Table 3).

**Table 3:** Correlation coefficient between the SABAS and NMP-Q, DSS, PHQ, and BSSS

Correlations							
			PHQ-9	DSS	NMP-Q	SABAS	BSSS
Spearman’s rho	PHQ	Correl. coefficient	1.000	0.388**	−0.347**	−0.349**	0.146*
		Sig. (two-tailed)	–	0.001	0.001	0.001	0.023
		*N*	240	240	240	240	240
	DSS	Correl. coefficient	0.388**	1.000	−0.541**	−0.595**	0.018
		Sig. (two-tailed)	0.001	–	0.001	0.001	0.779
		*N*	240	240	240	240	240
	NMP-Q	Correl. coefficient	−0.347**	−0.541**	1.000	0.626**	−0.039
		Sig. (two-tailed)	0.001	0.001	–	0.001	0.547
		*N*	240	240	240	240	240
	SABAS	Correl. coefficient	−0.349**	−0.595**	0.626**	1.000	−0.130*
		Sig. (two-tailed)	0.001	0.001	0.001	–	0.044
		*N*	240	240	240	240	240
	BSSS	Correl. coefficient	0.146*	0.018	−0.039	−0.130*	1.000
		Sig. (two-tailed)	0.023	0.779	0.547	0.044	–
		*N*	240	240	240	240	240

**Correlation is significant at the 0.01 level (two-tailed)

*Correlation is significant at the 0.05 level (two-tailed)

**Fig. 1:**
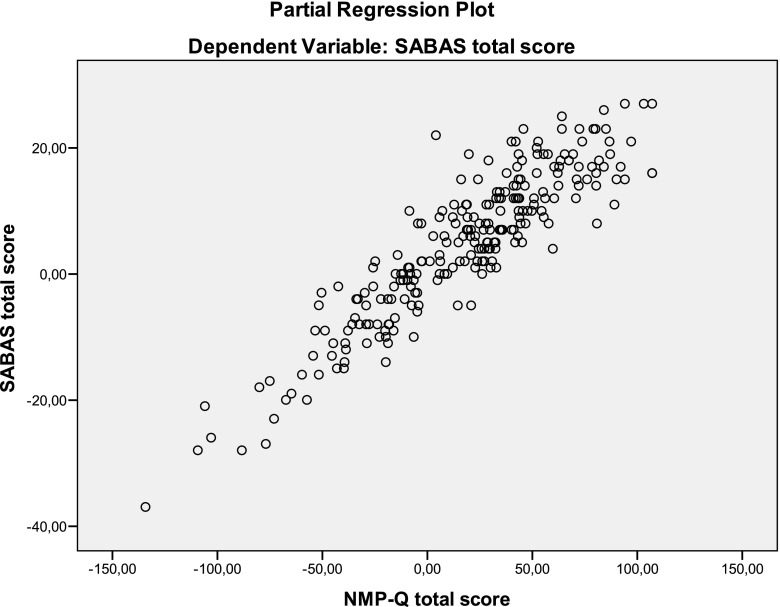
Linear regression between SABAS and NMP-Q score

The stepwise linear regression analysis, with SABAS score being the dependent variable and NMP-Q, DSS, PHQ, and BSSS being predictors, indicated that NMP-Q score was a significant predictor of SABAS score (*b* = 0.12, SE = 0.01, *p* < 0.01). Approximately 47% of the variance in SABAS was accounted for by all the predictors (*R* ^2^ = 0.47). Of this, the NMP-Q score explained 17.6% of the variance in SABAS (see Fig. 1).

The mean rank analysis between the two clusters of data (using a Mann-Whitney test) demonstrated significant differences in application use, such as traditional mobile communication, social media, and information gathering (see Table 4).

**Table 4:** The differences in application preferences between subjects of the two clusters

Application categories	Cluster	Number	Mean rank	*Z*	Asymp. sig. (two-tailed)
Traditional mobile communication	1	139	128.01	−2.46	0.014
	2	101	110.16		
Internet-based communication	1	139	114.92	−1.51	0.129
	2	101	128.17		
Social media	1	139	106.98	−3.68	0.001
	2	101	139.11		
Information	1	139	128.07	−2.61	0.009
	2	101	110.08		
Entertainment	1	139	123.45	−1.01	0.313
	2	101	116.45		
Games	1	139	120.86	−0.19	0.844
	2	101	120.00		
Directions and timetable	1	139	122.00	−0.90	0.365
	2	101	118.44		
Lifestyle applications	1	139	121.39	−0.43	0.66
	2	101	119.27		
Health-related applications	1	139	122.40	−1.43	0.150
	2	101	117.88		

ANOVA results show statistically significant differences between the test scores and the application categories (Table 5).

**Table 5:** The differences in application preferences with between subject’s test score

Application categories		SABAS	NMP-Q	DSS	PHQ-9	BSSS
Traditional mobile communication	*F*	2.82	5.50	2.26	2.06	1.41
	Sig.	0.039	0.001	0.081	0.105	0.239
Internet-based communication	*F*	2.32	1.64	3.40	0.45	1.37
	Sig.	0.075	0.180	0.018	0.716	0.250
Social media	*F*	8.40	5.10	3.35	1.26	0.78
	Sig.	0.001	0.002	0.020	0.287	0.505
Information	*F*	4.19	3.08	1.65	1.02	0.92
	Sig.	0.006	0.028	0.177	0.384	0.432
Entertainment	*F*	1.17	1.40	2.12	1.34	0.03
	Sig.	0.319	0.241	0.097	0.262	0.993
Games	*F*	0.30	0.66	0.67	0.21	0.96
	Sig.	0.822	0.576	0.570	0.889	0.409
Directions and timetable	*F*	0.39	0.31	0.40	0.31	0.87
	Sig.	0.756	0.812	0.752	0.815	0.453
Lifestyle applications	*F*	1.08	0.18	1.66	0.87	1.02
	Sig.	0.355	0.905	0.175	0.457	0.383
Health-related applications	*F*	1.26	0.40	0.22	0.46	0.61
	Sig.	0.287	0.748	0.878	0.705	0.605

## Discussion

In the present study, the psychometric properties of an English language version of a short form of smartphone addiction scale were assessed. The factor analysis of the six SABAS items identified one factor (i.e., a single component). By analyzing the psychometric characteristics of the SABAS, the analysis demonstrated good internal reliability and consistency of the scale. Congruent validity was also confirmed via its significant correlation with the NMP-Q and DSS. These results are in agreement with other scale validating studies regarding smartphone-related addiction supporting a one-factor construct alongside good reliability and validity.

The regression analysis of the data demonstrated that nomophobia, deprivation, depression, and sensation seeking predicted 47% of the total variance of the SABAS. In contrast to our findings, Jeong et al. (2016) found that sensation seeking was not a significant predictor of smartphone addiction. A possible explanation provided by other authors for the aforementioned association is that addictive symptoms among healthy populations do not reach pathological levels and that the patterns of addictive behaviors are not necessarily directly related to psychological constructs such as depression or sensation seeking (Billieux et al. 2016; Van Rooij and Prause 2014).

The present study found an inverse relationship between depression and excessive smartphone use, which is in accord with other studies suggesting that the active use of social applications may be beneficial in lowering depressive symptoms (Verduyn et al. 2017; Elhai et al. 2017a, b). A possible explanation for these results might be that depressive symptoms are not necessarily associated with social withdrawal and general avoidance of social interaction. Alternatively, smartphone use may contribute to increased social engagement and decrease feelings of loneliness and isolation (Kim et al. 2016a, b). Our results are in agreement with those of a representative Hungarian population of 5961 adolescents, which reported 4.5% at risk for social media use addiction, and association with low self-esteem, and high level of depression symptoms (Banyai et al. 2017).

The present study demonstrated significant differences in application use, such as traditional mobile communication, social media, and information gathering. According to the results, it can be argued that mobile communication, social media, and information gathering were the most relevant smartphone applications in influencing the scores obtained on the SABAS, NMP-Q, DSS, PHQ, and BSSS. The results here are in line with previous research, which found that social networking was one of the most popular online activities, followed by e-mail, chat, and videos and movies (Griffiths and Szabo 2014).

A limitation of the present study is that the research was conducted on a small healthy volunteer sample, rather than on a clinical one. Therefore, it is difficult to document the presence of other psychological deficiencies present in the participants’ lives. Furthermore, the analysis utilized self-reported cross-sectional data from a self-selected sample of participants, which might lead to distortions in results. The present authors cannot argue for a determinant category of symptoms manifesting along the excessive smartphone use. However, the study supports the existence of smartphone use-related addictive symptoms. Finally, the study does not provide any information regarding the harm and persistency of the problems assumed to be assessed through the new instrument. The study of the associations with other personality-related variables is recommended in further research.

## Conclusion

By validating an English version of the Hungarian version of the SABAS (Csibi et al. 2016), we provided a short, easy-to-use, and easy-to-score tool for screening the risk of smartphone addiction, with the potential of exceeding cultural boundaries. The study adds some empirical data concerning the possible core determinants of smartphone application-related addictive behavior without establishing a clear causality in the effects. The most highly ranked preferences for smartphone-based applications were communication, social media, and information gathering in the sample with relevant impact on SABAS and NMP-Q scores. Significant associations were found between these choices and scores reported on deprivation sensations, nomophobia, and SABAS, alongside a negative significant correlation with depression and sensation seeking variables.

## Appendix 1

**Smartphone Application-Based Addiction Scale (SABAS)**

Please indicate the extent to which you agree or disagree with the statements below in relation to your smartphone use habits.

**Table:**

	Strongly Disagree	Disagree	Slightly Disagree	Slightly Agree	Agree	Strongly Agree
My smartphone is the most important thing in my life.	1	2	3	4	5	6
Conflicts have arisen between me and my family (or friends) because of my smartphone use.	1	2	3	4	5	6
Preoccupying myself with my smartphone is a way of changing my mood (I get a buzz, or I can escape or get away, if I need to).	1	2	3	4	5	6
Over time, I fiddle around more and more with my smartphone.	1	2	3	4	5	6
If I cannot use or access my smartphone when I feel like, I feel sad, moody, or irritable.	1	2	3	4	5	6
If I try to cut the time I use my smartphone, I manage to do so for a while, but then I end up using it as much or more than before.	1	2	3	4	5	6

The total score is calculated by adding the individual scores (1-6) of each item (providing a minimum score of 6, and a maximum score of 36)

## Footnotes

1 Nomophobia: a specific phobia caused by the anxiety of the lack of availability of a smartphone or other communication device (after Yildirim and Correia 2015).
